# A Conversation with Julie Cosmidis

**DOI:** 10.1021/acscentsci.2c00980

**Published:** 2022-09-01

**Authors:** Carolyn Wilke

A world away, the *Perseverance* rover is traversing the Martian surface and stashing samples of rock. Planetary scientists can’t wait to get their hands on that cache, which NASA hopes to bring to Earth in the early 2030s. In the meantime, Julie Cosmidis is warning her colleagues not to jump to conclusions based on very limited data—because chemistry can create near copies of life without any help from biology.

**Figure:**
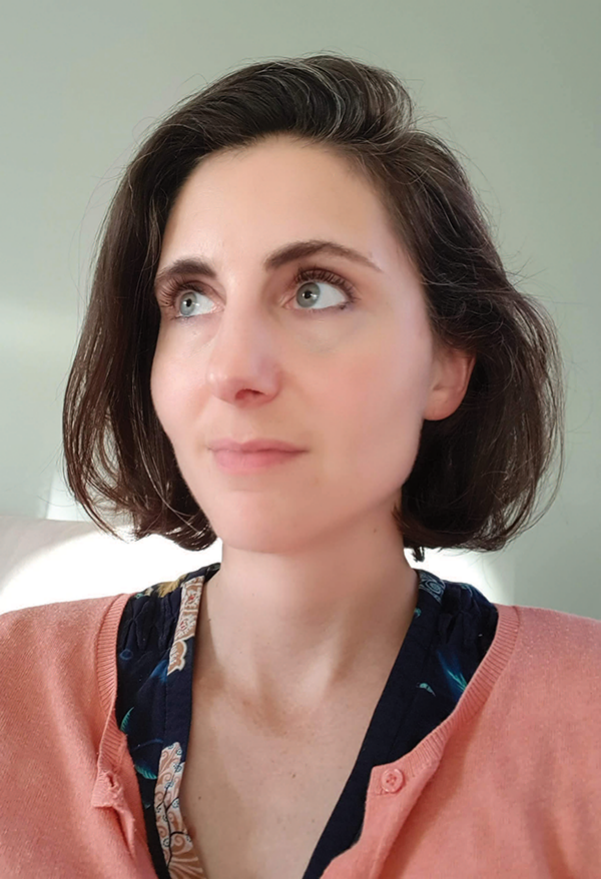
Credit: Peter Knight

In a review published last year, Cosmidis, who studies biominerals at the University of Oxford, and coauthor Sean McMahon of the University of Edinburgh shared how the history of the search for life on early Earth and on other planets has been littered with blunders. They cited errors as far back as the 1800s, when sinewy filaments observed in rocks were mistaken for ancient organisms. Cosmidis’s lab works to identify how certain geological systems can create other false signs of life, and she wants biologists to realize just how common those can be.

Carolyn Wilke spoke to Cosmidis about the ways chemistry can create biological mimics and about the methods researchers can use to scour rocks for relics of life. This interview was edited for length and clarity.

## How did you start looking for structures that resembled living things?

I’m not an astrobiologist really. I’m much more of a geomicrobiologist. During my postdoc at the University of Colorado Boulder, I was working on bacteria that produce sulfur minerals, and I was using a growth medium that contained both sulfide and organic molecules. The microbes grew very well, and then I also had to do abiotic control experiments. This helps you see what’s happening in your medium, just with chemistry and without any influence of life.

We noticed that we were growing sulfur minerals in these controls. When we put them under the microscope and discovered these carbon–sulfur structures with these crazy spherical and filamentous morphologies. Just in morphology, they really look like bacteria.

So I spent weeks and weeks reproducing the experiment, checking that there was no contamination. And there was nothing; it was really chemistry. They were so fascinating in the way they were mimicking life.

## Why are you concerned that researchers looking at samples from Mars might be fooled by false signs of life?

Lots of chemical processes, mostly when molecules and structures self-organize, can create objects that look like they could have been formed by life. These processes have been discovered by chemists who were working on self-organization for totally different reasons, so the astrobiology community is not always aware of these things.

A 1996 *Science* paper described this meteorite and objects inside it that looked like fossil bacteria. It was a huge discovery because it was the first trace of life outside Earth. But it took several years to find out that they were not bacteria. There were papers and papers of scientific debates—so a lot of resources and press attention—for nothing. We should avoid making the same errors.

## What do false signs of life often look like, and how do they form through chemistry?

When you precipitate minerals in the presence of organics, they tend to form rounded shapes. It is easy to create spheres, which a lot of people are after when they look for life in the rock record because there are so many spherical bacteria.

We call these things that look like biology, but are not, “biomorphs.” The term was coined by a group led by Juan Manuel Garcia-Ruiz.

My group found a biomorph-forming process: if you react organic molecules with sulfide, which is very abundant on Mars and in some environments of the early Earth, they form spherical and tubular objects that have an organic envelope. We found that if you try to fossilize them in silica, they will be preserved better than bacteria are because the biomorphs are filled with sulfur, which is a hard mineral.

So morphological preservation is one thing; the second thing is chemical preservation. During fossilization, sulfur is incorporated in the organic molecules. This makes it more recalcitrant. You would be more likely to find these objects preserved in the rocks than to find actual bacteria.

**Figure:**
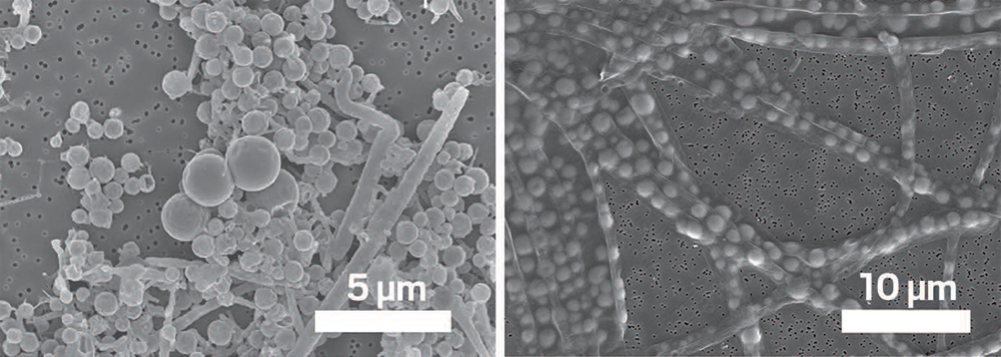
Cosmidis’s team found that, reacting a sulfide solution with a mix of organics produced biomorphs, spheres that look like and even have a similar nitrogen-to-carbon ratio as bacteria (left). In contrast, in an experiment mimicking fossilization of real spherical microbes, these Thiotrix bacteria were flattened and lost their spherical shape (right). Credit: Christine Nims.

## What other signs of life can be faked by chemistry?

We are also looking at chemical signals. Some are just the presence of organic matter. You can find lots of organic matter in space, in meteorites and comets. And you can also have organic matter created by hydrothermal processes. We are interested in the isotopic composition of this organic matter, and we know that different biological processes can fractionate isotopes in a specific way. But for each of these proposed isotopic biosignatures, you can find a chemical process that fractionates isotopes the same way.

For example, enzymes in the photosynthetic process make organic matter that is lighter in isotopic composition than the CO_2_ it was derived from. So we often say that organic matter with slightly lower levels of carbon-13 is likely to be biological because that’s what photosynthesis does. The problem is that hydrothermal processes can also produce organic matter with depleted carbon-13 levels, too.

Similarly, some microbial metabolisms of sulfur-cycling bacteria fractionate sulfur isotopes in a certain way, but there are photochemical processes in the atmosphere that can do the same thing.

## So what are some best practices?

If you take what we think are biosignatures one by one, they can all be recreated by chemistry. If you find an object that combines them all, you increase the likelihood that it’s the real thing. The problem is we’re not ready to do that for Mars because these rovers can’t measure everything the way we would on Earth with all the analytical methods we have available.

In a decade, we will be able to do a very in-depth investigation using many techniques when we have samples from Mars. With position-specific isotopic measurements, you can know the isotopic ratios at specific carbons in a molecule. This signature is more specific to the process that creates the molecule. I think that is going to be a powerful method when we are able to implement it on extraterrestrial samples.

There should also just be more discussion between chemists and a lot of people working in self-assembly in totally different contexts. Having more communication between these communities and astrobiology would be great.

## Carolyn Wilke is a freelance contributor to

*Chemical & Engineering News*, *an independent news publication of the American Chemical Society.*

